# School Variation in Asthma: Compositional or Contextual?

**DOI:** 10.1371/journal.pone.0008512

**Published:** 2009-12-30

**Authors:** Tracy K. Richmond, S. V. Subramanian

**Affiliations:** 1 Division of Adolescent Medicine, Children's Hospital Boston, Boston, Massachusetts, United States of America; 2 Department of Society, Human Development, and Health (SVS), Harvard School of Public Health, Boston, Massachusetts, United States of America; University of Pittsburgh, United States of America

## Abstract

**Background:**

Childhood asthma prevalence and morbidity have been shown to vary by neighborhood. Less is known about between-school variation in asthma prevalence and whether it exists beyond what one might expect due to students at higher risk of asthma clustering within different schools. Our objective was to determine whether between-school variation in asthma prevalence exists and if so, if it is related to the differential distribution of individual risk factors for and correlates of asthma or to contextual influences of schools.

**Methods:**

Cross-sectional analysis of 16,640 teens in grades 7–12 in Wave 1 (data collected in 1994–5) of the National Longitudinal Study of Adolescent Health. Outcome was current diagnosis of asthma as reported by respondents' parents. Two-level random effects models were used to assess the contribution of schools to the variance in asthma prevalence before and after controlling for individual attributes.

**Results:**

The highest quartile schools had mean asthma prevalence of 21.9% compared to the lowest quartile schools with mean asthma prevalence of 7.1%. In our null model, the school contributed significantly to the variance in asthma (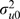 = 0.27, CI: 0.20, 0.35). Controlling for individual, school and neighborhood attributes reduced the between-school variance modestly (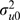 = 0.19 CI: 0.13–0.29).

**Conclusion:**

Significant between-school variation in current asthma prevalence exists even after controlling for the individual, school and neighborhood factors. This provides evidence for school level contextual influences on asthma. Further research is needed to determine potential mechanisms through which schools may influence asthma outcomes.

## Introduction

Asthma is the most common chronic disease of childhood with the most recent U.S. national prevalence estimates at 13% [1]. Over the last two decades the rapid increase in U.S. asthma prevalence and morbidity–with substantial associated U.S. healthcare costs–has made childhood asthma an even more pressing public health problem both in the U.S and abroad [2]. The increasing prevalence has exacerbated existing racial/ethnic and socioeconomic disparities in U.S. asthma related outcomes with nonwhite American children living in urban areas and/or in poverty being disproportionately affected [3].

To date, asthma research has largely focused on and succeeded in identifying individual risk factors for asthma such as allergen exposure, tobacco exposure, access to/utilization of health care, socioeconomic status, or race/ethnicity [6]–[12]. Though these risk factors are not uniformly distributed across any population and may partially explain the etiology of U.S. based disparities in asthma outcomes, changes in individual risk factors are incapable of fully explaining the disproportionate rise of asthma in certain U.S. groups. Despite this, until recently, few U.S. based studies looked to environmental contexts for potential explanations for the worsening disparities in asthma outcomes [13].

Recent U.S. and international studies have demonstrated neighborhood level variation in asthma outcomes [14]–[16]. Differences in air quality, violence exposure, and/or allergen exposure have been posited as explanations for between neighborhood differences in asthma outcomes [14], [17]–[18]. Others have examined the role of neighborhood level socioeconomic factors on asthma and have found inconsistent results [19]–[21].

Schools are likely to influence childhood asthma outcomes through mechanisms similar to those proposed for neighborhoods. Numerous U.S. based and European studies have demonstrated considerable allergen loads in school environments and have linked school-based exposure to individual students' asthma related outcomes [22]–[30]. However, we are aware of no studies that have examined U.S. school related asthma outcomes from a multilevel perspective. A multilevel analytic approach provides an opportunity to evaluate the school differences due to compositional effects (i.e. more students with risk factors for asthma cluster within certain schools) versus contextual effects (i.e. the school has an effect on asthma outcomes above and beyond that expected due to the asthma risk profile of the student body) [31]. In this study, we use a nationally representative U.S. school based study of adolescents to determine if differences in asthma prevalence between U.S. schools exist and if so, if they are due to the individual risk profiles of students or to a contextual effect of the schools.

## Methods

### Subjects

This research uses data from the first of four Waves of the National Longitudinal Study of Adolescent Health (Add Health), a nationally representative U.S. school-based study of adolescents enrolled in grades 7 through 12. Wave I data at the individual, family, school, and community level were collected between 1994 and 1996, Wave II data one year later, Wave III data from 2001 to 2002, and Wave IV data in 2008 [32]. The primary sampling unit of the Add Health study is schools; schools were stratified by size, type (private v. parochial v. public), census region, level of urbanization, and percentage of students who are white prior to sampling to ensure a representative sample of U.S. schools. All students were asked to complete the In-School questionnaire. A subset of students was sampled after stratifying by grade and gender and asked to complete the In-Home survey. The final In-Home sample used here was derived from 132 participating schools with approximately 200 students per school.

Parents of In-Home participants were also asked to complete a questionnaire. Mothers or other female heads of households were the preferred respondents. Only if a female head of household was unavailable (i.e. there was none living in the home) was the father or other male parental figure interviewed. In addition to the In-Home Survey and Parental Questionnaire, this study uses data from the School Administrator Survey which was administered to school administrators of all participating schools.

Our outcome variable was taken from a response to the Parental Questionnaire regarding asthma in their child. Our overall sample size thus was largely dependent on the response rate to the Parental Questionnaire; approximately 15% of participants in the In-Home Survey did not have a parent who completed the Parental Questionnaire. We excluded 3,012 participants who had missing data for the dependent variable as well as those who had data missing for more than 3 of the 13 independent variables. Our measures of socioeconomic status were also derived from the Parental Questionnaire and thus, had a similar non-response rate to that of our outcome variable. In an effort to avoid selection bias and inaccurate inferences resulting from listwise deletion of those who were missing socioeconomic measures but not the outcome variable, we imputed the SES measures—reported household income and education level of mother– by best-subset regression [33]–[34]. After this imputation and all exclusions, our final sample contained 14,191 adolescents nested within 132 U.S. schools.

### Study Variables

#### Outcome variables

Our outcome variable was parental report of whether the child (our study participant) currently had asthma. The parent was asked “For each of the following health problems, please tell me if (child/study participant) has it now. Also tell me whether his/her biological mother and/or his/her biological father has it now.” The list of conditions included asthma/emphysema. We dichotomized this variable into a yes/no variable and treated the responses “don't know” and “refused” as missing.

#### Independent individual variables

Demographic variables controlled for included age, gender, race/ethnicity, U.S. nativity, maternal education, and household income, all variables that have been found to be associated with asthma in prior studies. Age and gender were self-reported by the participants. Race/ethnicity was constructed from two questions, one which asked participants to indicate if they were of Hispanic/Latino origin and the second which asked them to choose a category of race that best describes them. We constructed 6 mutually exclusive categories: Hispanic, Black or African-American (not Hispanic), Asian/Pacific Islander, White (not Hispanic), Native American/American Indian, and Other. U.S nativity was ascertained from a single question asking the respondent whether or not they were born in the U.S.

Socioeconomic measures were taken from the Parental Questionnaire. Parents were asked to report household income over the last year in U.S. dollars. We transformed the household income measure into a measure relative to the U.S. poverty level by taking into consideration the household size then comparing it to U.S. poverty thresholds in 1995, the year the data were collected [35]. The parent respondent was asked to report the highest level of education achieved by the participant's mother. Responses were categorical and ranged from no school to professional school. We dichotomized this measure into no high school diploma versus high school degree and beyond. As mentioned above, there was significant missing data for maternal education and household income reflecting the response rate to the Parental Questionnaire and so we present findings using the imputed values.

We controlled for additional variables including insurance status, ease of accessing health care, Body Mass Index (BMI), the presence of smokers in the house, and parental asthma status all of which are potential risk factors for asthma. We chose to include variables describing the individual's insurance status and ease of accessing health care in an effort to reduce bias resulting from undiagnosed asthma due to obstacles to seeking care. We included BMI as it has been shown in other studies to be associated with asthma. Additionally, both the presence of smokers in the house and having a parent with asthma increases the child's risk of asthma. All except Body Mass Index relied on parental response and were yes/no variables. BMI was constructed using participant reported height and weight.

#### Additional variables

In an effort to produce a more conservative estimate of the between school variance we controlled for variables both at the school level as well as variables describing the neighborhood in which the participant resided. We viewed the inclusion of two neighborhood demographic variables as a crude sensitivity analysis used to evaluate whether school contexts were acting merely as a proxy for neighborhood influences. We included two variables to describe the demographics of the school: the school level median household income and the percentage of the student body who are White. The school level median household income was constructed as a composite of the household income reported by the parents of individual students attending the same schools. The percentage of the student body that is White was reported by the school administrators in categories (0%, 1–66%, 67–93%, 94–100%). We dichotomized this variable to less than or equal to 66% and greater than 66%. In an additional model we included two markers of the neighborhood sociodemographics taken from Census data: the proportion of the neighborhood who are White and the median household income of the neighborhood. Finally, we added region of the country to account for any geographic differences in asthma prevalence.

### Data Analysis

We first examined bivariate relationships between variables of interest and current asthma status. We then used multilevel statistical modeling techniques to partition the variance in current asthma prevalence between the school and individual levels [36]–[37]. The substantive relevance of these models have been well discussed [38]–[40]. Specifically, two-level models were estimated, with a dichotomous response, *y*, currently has asthma or not, for individual 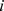 attending school 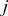 of the form: 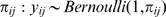. In our null model, the probability, 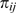, was related to an overall mean and a random effect of the school, by a logit link function as logit (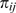) = 
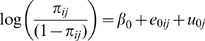
. The parameter 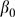 estimates the mean log odds of having asthma across the sample while the parameter 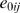 represents the random differential at the individual level and 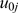 represents the random differential from the mean at the school level. These differentials are each assumed to have an independent and identical distribution and variances (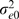, and 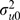, respectively). The variance estimate for the null model estimates the unconditional or unadjusted variation in asthma prevalence that exists *between* schools.

We then added individual level variables to the null model as: 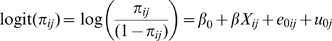 in which 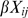 represents the coefficient associated with individual level variables. We re-estimated the between school variance (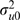) after adjusting for the individual covariates in order to ascertain the variance that remains after controlling for the composition of the school. We then added two school-level variables, the median household income at the school level and a measure of the racial/ethnic composition of the student body in the form of: 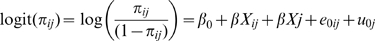 where 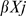 represents the coefficient of the school level variables. In further models, we added two variables describing the home neighborhood of the student participant, the median household income of the neighborhood and the percentage of the neighborhood population that is White and finally variables representing the geographic regions of the country. Estimates are reported from the logistic models using the xtlogit algorithm as implemented with STATA 10 [41]–[42].

## Results

Table 1 provides the frequency of individual and school level factors by asthma status. Parent reported current asthma differed by race/ethnicity with Blacks (12.7%) and Native Americans (16.1%) having the highest reported occurrence and Asians (8.9%) and Mexicans (7.3%) having the lowest. In Table 2, we see that schools differed considerably relative to their mean asthma prevalence. After dividing schools into quartiles based on their asthma prevalence, we see that those in the highest quartile had mean asthma prevalence of 22% while those in the lowest had mean asthma prevalence of 7%.

**Table 1:** Participant characteristics by asthma status.

	No Asthma	Asthma	p-value
**Ethnicity**			**<0.001**
White	88.2%	11.8%	
Black	87.3%	12.7%	
Native American	83.9%	16.1%	
Asian/PI	91.1%	8.9%	
Other	88.1%	12.0%	
Non-Mexican Hispanic	89.9%	10.1%	
Mexican	92.7%	7.3%	
**Age**	16.1 (1.7)	16.0 (1.7)	**<0.001**
**Gender**			0.060
Female	88.8%	11.2%	
Male	87.9%	12.1%	
**BMI (mean)**	22.4 (4.4)	22.9 (4.8)	0.99
**Household Income (mean)**	$45,554	$46,917	0.87
**Maternal Education**			**<0.001**
Less than High School graduate	17.1%	13.7%	
High School graduate +	82.9%	86.3%	
**Nativity**			**<0.001**
U.S. Born	87.9%	12.1%	
Foreign born	94.5%	5.5%	
**Tobacco Use**			**0.043**
Yes	87.5%	12.5%	
No	88.7%	11.3%	
**Exposure to smoke**			**<0.001**
No smokers in home	89.4%	10.6%	
Smokers in home	87.1%	12.9%	
**Maternal Asthma**			**<0.001**
Yes	67.7%	32.3%	
No	90.3%	9.8%	
**Paternal Asthma**			**<0.001**
Yes	65.6%	34.5%	
No	90.0%	10.0%	
**Accessibility of healthcare**			0.36
Easy to access	88.2%	11.8%	
Difficult to access	88.9%	11.1%	
**Insurance status**			**0.015**
Insured	88.1%	11.9%	
Not insured	89.9%	10.1%	
**School level median household income**	$40,163	$41,275	0.99
**Percentage of student body who is white**			0.26
0%	87.9%	12.1%	
1–66%	88.6%	11.4%	
67–93%	88.8%	11.2%	
94–100%	87.6%	12.4%	

**Table 2:** Characteristics of schools defined by quartile of asthma prevalence.

School Asthma Prevalence Quartile	Quartile 1	Quartile 2	Quartile 3	Quartile 4	p-value
School level asthma prevalence	7.1%	10.3%	14.8%	21.9%	**<0.001**
**School racial/ethnic makeup**					**<0.001**
0% White	12.2%	4.9%	12.2%	13.8%	
1–66% White	49.5%	38.2%	20.9%	53.6%	
67–93% White	21.2%	40.4%	28.3%	19.9%	
94–100% White	17.1%	16.5%	38.6%	12.7%	
**School median household income**	$38,261	$40,883	$41,365	$40,656	**<0.001**

The results from a taxonomy of models starting with the null or empty variance component model and adding individual, school, neighborhood, and region variables are shown in Table 3. In the null or empty variance component model, the variance of the random effects of schools was significantly different from zero (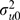 = 0.27, p<0.05). The addition of the individual-level variables to the fixed part of the model reduced the variance at the school level only modestly and it remained significantly different from zero (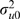 = 0.23, p<0.05). In this model, Blacks (OR = 1.19, p = 0.020) and Hispanics (OR = 1.27, p = 0.031) had on average higher odds of having asthma currently asthma than Whites; no other racial/ethnic group differed significantly from Whites in their odds of having asthma currently. On average, those who were born in the U.S. were found to be more likely to have asthma (OR 1.87; p<0.001) as were those who had smokers in their household (OR 1.18; p = 0.003) and who had a mother (OR = 4.09, p<0.001) or father with asthma (OR = 4.58, p<0.001) all other factors being held constant.

**Table 3:** A taxonomy of models examining the relationship between individual and school variables and student asthma status.

	Multilevel Model				
	M1	M2	M3	M4	M5
***Fixed Effects***					
*Intercept*	−2.02***	−2.51***	−2.73***	−2.78***	−2.58***
***Individual level variables***					
***Race/ethnicity*** ^					
*Black*		1.19*	1.17∼	1.08	1.11
*Asian*		0.92	0.87	0.79	0.75∼
*Native American*		1.3	1.34	1.39∼	1.34
*Other*		0.85	0.84	0.84	0.97
*Non-Mexican Hispanic*		1.27*	1.23*	1.26*	1.24∼
*Mexican*		0.83	0.77∼	0.77∼	0.73*
***Age***		0.94**	0.94***	0.94**	0.94**
***Female gender***		0.91∼	0.91∼	0.89*	0.89*
***Body Mass Index***		1.02**	1.02**	1.02**	1.02**
***U.S. Nativity***		1.83***	1.78***	1.71***	1.69***
***Household income as percent poverty***		1.01	1.01	1.01	1.01
***Mom with less than hs educ***		0.87	0.9	0.88	0.88
***Have insurance***		1.12	1.08	1.08	1.06
***Difficulty accessing care***		1.06	1.07	1.07	1.07
***Smokers in the house***		1.18**	1.20***	1.21***	1.22***
***Mom with asthma***		4.09***	4.13***	4.15***	4.12***
***Dad with asthma***		4.58***	4.52***	4.58***	4.56***
***School-level variables***					
***>66% of student body is White***			0.87∼	0.95	0.98
***Median household income of students***			1.14***	1.07	1.06
***Neighborhood-level variables***					
***Percent of population that is White***				0.75∼	0.77
***Median household income***				1.00*	1.00*
***Region*** #					
***South***					0.76**
***Midwest***					0.80*
***Northeast***					0.91
*Random Effects*					
***sigma_u***	0.27*	0.23*	0.21*	0.20*	0.17*
***rho***	0.021***	0.016***	0.0096***	0.012***	0.0089***
*Goodness of Fit*					
***log likelihood***	−6145.9	−4822.7	−4700.5	−4526.1	−4522

Key: ∼p<0.10; *p<0.05; **p<0.01; ***p<0.001.

∧ White is the reference group.

# West is the reference group.

We next sequentially added variables describing the school, the neighborhood in which the participant resides, as well as the region of the country in which the participant lives. When we controlled for individual and school level variables only, we found that students who attended schools with a greater percentage of white students had decreased odds of having asthma (OR = 0.87, p = 0.073) but that those students who attended schools with higher median household incomes had on average higher odds of having asthma. However, when we controlled for neighborhood attributes as well, neither school variable was predictive of individual asthma status in our population. Controlling for school and neighborhood attributes also attenuated the association between Black and Hispanic race/ethnicity and asthma status. Finally we controlled for the region of the country in which the student participant lived and found that those living in the South and the Midwest had significantly reduced odds of having asthma when compared to those living in the West.

**Figure 1:**
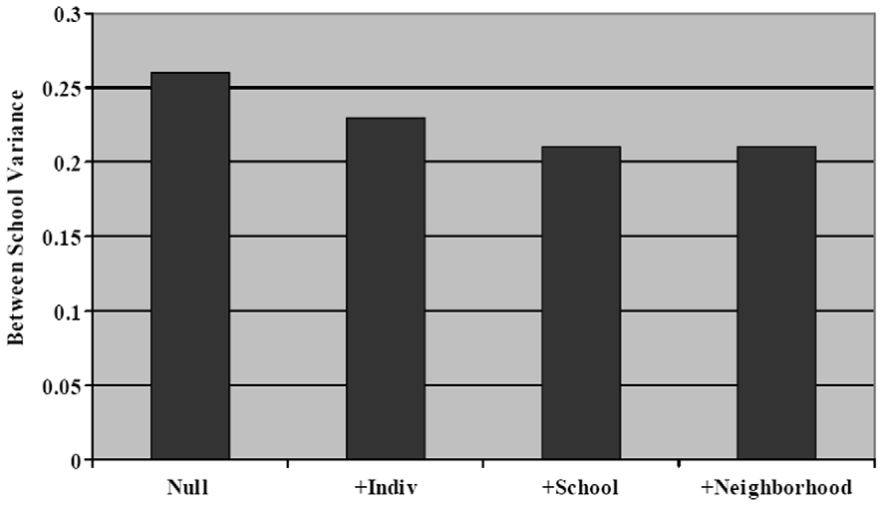
Between school variance remains even after controlling for compositional effects and contextual descriptors.

Figure 1 graphically depicts the change in between-school variance with the sequential addition of individual, school, and neighborhood level correlates and risk factors for asthma. The between-school variation remains significant and is only modestly reduced after controlling for additional variables.

In an effort to insure that our results were not reliant on a few schools with extreme values of average asthma prevalence we performed sensitivity analyses. We examined the distribution of the school-level average asthma and excluded those schools whose asthma prevalence was >98^th^ percentile (schools with >38% of student body with asthma). For a second sensitivity analysis we excluded schools with sample size less than 10. In both instances–excluding schools that seemed extreme on our outcome or potentially influential due to small sample size—there was no substantive difference in our models.

## Discussion

This study investigates to what extent *between* school differences in asthma prevalence exist and whether between school differences are attributable to an unequal distribution of individual risk factors and asthma correlates in certain schools. We find that the differential distribution of students at higher risk of asthma does not completely explain between school differences. Instead, we find evidence for a contextual effect of schools on asthma prevalence. To our knowledge this is the first study to explore the contextual influence of schools on adolescent asthma prevalence in a large nationally representative U.S. school based population.

Our findings at the individual level largely mirror those of other studies. Non-Mexican Hispanics and Blacks, those born in the U.S. and those with either a mother or father with asthma were noted to have higher prevalence of asthma. Interestingly, however, in our analysis neither marker of individual SES—household income nor maternal education level—was predictive of asthma. These findings speak to the inherent complexity in the social patterning of asthma prevalence.

Our findings add to the growing body of literature demonstrating school-level differences in health related behaviors and outcomes [43]–[46]. School level differences in academic achievement independent of student demographics have long been noted [43], [47]. More recently studies have demonstrated between school differences in health related behaviors such as tobacco and alcohol use [44], [48], [49], and weapon carrying [50], [51] as well as our own work on physical activity [52], all of which may be partially attributable to school level norms, policies, or opportunities. Though the literature linking school environments to health related outcomes is growing, this is one of very few studies to demonstrate a school contextual effect on a health *outcome* [53].

Due to the limitations in our data regarding structural, cultural, or other differences in schools, we were unable to explore possible mechanisms through which schools are influencing students' asthma status. However, we hypothesize that schools may influence students' asthma outcomes through several different pathways. First, schools may differ in their allergen exposures for students. Numerous studies have demonstrated high levels of allergens in schools including mold, cat, dog, mouse, dust mite, and cockroach allergen as well as high exposure to VOCs [22]–[26], [54]. Several European studies have linked increased exposure to specific allergens in schools to worse asthma related outcomes in both students and teachers [27]–[30], [55]. Because students spend such a significant portion of their waking hours in school and the link between allergen exposure and asthma related outcomes is so well documented, it is logical to think that schools with higher level of allergen exposure would have on average students with higher asthma prevalence.

Schools may also influence the asthma prevalence and morbidity in students through their physical structures. Studies by the Government Accounting Office have shown that the physical structures of schools vary and specifically vary by the demographics of the student body [56]. Schools with high percentages of low-income and/or racial/ethnic minority students are more likely to report that the school building has an unsatisfactory environmental condition such as poor ventilation. Students attending such schools may potentially have on average worse asthma related outcomes.

Finally, the stress related school environment may influence asthma related outcomes in students. Stress and more specifically exposure to violence have been increasingly recognized as risk factors for worsening asthma related outcomes [57]–[64]. School environments may expose students to stress through school-based violence, racial/ethnic or socioeconomic tension such as racism, and/or through high academic or social demands. Because stress is likely distributed unevenly between schools, we hypothesize that it may contribute to between school differences in asthma.

Because the schools were not nested within communities (i.e. students from more than one community might attend the same school and students from a single community might attend more than one school), we were unable to tease apart the effects of schools versus communities. It is noteworthy however, that when we controlled for the socioeconomic and racial/ethnic makeup of the neighborhood of residence of the students, that the between school variance was altered very little. Though we recognize that schools, especially public schools, are largely influenced by the neighborhoods in which they exist, there may be substantial differences in exposures between a students' home and school which make the question of neighborhood versus school influence on asthma worthy of pursuit.

There are several limitations to this paper that must be acknowledged. First, these data were collected more than 10 years ago and may not be reflective of the current situation among schools. However, the prevalence of asthma has continued to rise over the last decade and the social patterning of asthma has become amplified [3]. At the same time, the physical structures as well as social norms in schools remain non-uniform. Thus our findings may underestimate the current situation. Given the longitudinal nature of the data, it is imperative to perform analyses on baseline data in order to understand factors that may influence the health of adolescents as they transition into young adulthood and beyond. This study lays the groundwork for our future work using additional waves of Add Health. Another limitation to these analyses is our reliance on parental reported asthma status of the child. We do not have further data on severity of illness, recent exacerbations, or hospitalizations that would help us obtain a more nuanced understanding of asthma related outcomes beyond current prevalence. However, this is a problem commonly confronted in asthma studies. Additionally, the parent is asked if the child has the condition *now*. Thus, we have no information about the chronicity of the condition nor how it relates to the time attending the current school.

In conclusion, we find significant variation in asthma prevalence between schools that cannot be explained by the racial, socioeconomic or clustering of other asthma risk factors in the student body. This implies that the school has a contextual influence on asthma outcomes independent of the makeup of the student body. This has significant public health implications in that schools should be an area of focus in trying to improve all asthma outcomes and to eliminate the disparities currently seen.
